# Psychological Distress and Coping amongst Higher Education Students: A Mixed Method Enquiry

**DOI:** 10.1371/journal.pone.0115193

**Published:** 2014-12-15

**Authors:** Christine Deasy, Barry Coughlan, Julie Pironom, Didier Jourdan, Patricia Mannix-McNamara

**Affiliations:** 1 Department of Nursing and Midwifery, University of Limerick, Limerick, Ireland; 2 Department of Psychology, University of Limerick, Limerick, Ireland; 3 ACTé, ESPE Clermont-Auvergne, Université Blaise Pascal, Clermont-Ferrand, France; 4 Department of Education and Professional Studies, University of Limerick, Limerick, Ireland; Federal University of Rio de Janeiro, Brazil

## Abstract

**Background:**

Psychological distress among higher education students is of global concern. Students on programmes with practicum components such as nursing and teacher education are exposed to additional stressors which may further increase their risk for psychological distress. The ways in which these students cope with distress has potential consequences for their health and academic performance. An in-depth understanding of how nursing/midwifery and teacher education students experience psychological distress and coping is necessary to enable higher education providers to adequately support these students.

**Methods:**

This mixed method study was employed to establish self-reported psychological distress (General Health Questionnaire), coping processes (Ways of Coping Questionnaire) and lifestyle behaviour (Lifestyle Behaviour Questionnaire) of a total sample (n = 1557) of undergraduate nursing/midwifery and teacher education students in one university in Ireland. Individual interviews (n = 59) provided an in-depth understanding of students experiences of psychological distress and coping.

**Results:**

A significant percentage (41.9%) of respondents was psychologically distressed. The factors which contributed to their distress, included study, financial, living and social pressures. Students used varied coping strategies including seeking social support, problem solving and escape avoidance. The positive relationship between elevated psychological distress and escape avoidance behaviours including substance use (alcohol, tobacco and cannabis) and unhealthy diet is of particular concern. Statistically significant relationships were identified between “escape-avoidance” and gender, age, marital status, place of residence, programme/year of study and lifestyle behaviours such as diet, substance use and physical inactivity.

**Conclusion:**

The paper adds to existing research by illuminating the psychological distress experienced by undergraduate nursing/midwifery and teacher education students. It also identifies their distress, maladaptive coping and the relationship to their lifestyle behaviours. The findings can inform strategies to minimise student distress and maladaptive coping during college and in future professional years.

## Introduction

The mental health and wellbeing of young people is of global concern [1], [2], particularly in Ireland where it has been identified as a significant issue [3]. Significant levels of psychological distress have been reported in higher education students globally [4]–[9], who experience greater psychological distress than the general population [10]–[14]. Gender differences in psychological distress are evident with females reporting more psychological distress than males [5]–[8], [15], [16]. However; some caution is needed when examining the evidence on psychological distress because international study comparisons are difficult. This is due to a number of factors not least of which are the variances of prevalence of reported psychological distress, the instruments used to measure the distress and the cut off points used by the researchers to determine psychological distress.

Psychological distress has been widely used as an indicator of mental health [17]. Researchers such as Horwitz [18] consider it a transient emotional response to stress, which if untreated is pathological resulting in depression. Others such as Wheaton [19] argue that psychological distress is a relatively stable condition which impacts on social functioning and day-to-day living. There is general consensus in the literature that psychological distress is an emotional state characterised by symptoms of depression and anxiety [20]. The authors concur with Horowitz that psychological distress if left untreated can have deleterious impact upon mental health and wellbeing.

Psychological distress is important from a health promotion/illness prevention perspective because of its links with risk behaviours and physical illness in higher education students [21] and its propensity to precede more serious mental health disorders [18], [22].It is also of concern to education providers because of its negative impact on student learning [12]. As psychological distress is experienced in response to stress and is associated with a perceived inability to cope effectively [23] its relationship with stress and coping among higher education students is also of interest.

Stress, which is acknowledged as part of the student experience [24], has been defined by Lazarus and Folkman [25] as ‘a particular relationship between the person and the environment that is appraised by the person as taxing or exceeding his or her resources and endangering his or her well-being’ (p. 19). Situations or events that are perceived as threats to well-being and exceeding available coping resources tend to be experienced as stressful. Stress can be positive in that it challenges and motivates students to achieve, however it can also adversely impact upon student learning [26], their judgment and adaptive functioning [27]. Stress can lead to health damaging behaviours [28], [29] and is associated with negative physical health outcomes, the exacerbation of mental health symptoms and psychological distress [12], [30], [31]. Indeed stress has been identified as a significant predictor of psychological distress among higher education students [32], [33]. Stressors for higher education students have been identified as including lack of financial certainty, poor employment prospects, increased pressure to do well and technological overload [27]. Programmes of study with a substantial practicum component have been established as significantly stressful for undergraduate students. Nursing students are recognised internationally as a student cohort who experience high levels of stress [34], [35] and psychological distress [33], [36]. Similarly teacher education students report a good deal of stress during their programmes of study [37]–[39] and also report psychological distress particularly in relation to practicum [37], [40].

Coping is established as a key variable in the process of reducing, minimising or tolerating stress [41] and in preventing psychological distress [42]. Coping is defined as cognitive and emotional attempts to deal with the internal or external demands of the encountered situation [43]. It is perceived as a process as opposed to a trait or outcome. Higher education students cope in different ways with varying levels of success. For a minority of students the challenge is too great, and consequently they exit their programmes of study [44]. Some adopt positive methods such as seeking social support [3], [45] or using leisure activities [46] while others use maladaptive strategies (e.g. escape/avoidance) to manage stress [47]. The poor coping reported by many third level students in Ireland, including ignoring the problem, failure to seek help from others and escapism through substance use [8] is of concern, particularly in light of the findings that suicide ideation, self-harm and suicide attempts are higher in young people who do not seek help or talk about their problems [3].

The literature indicates that student nurses use diverse coping strategies including seeking social support [48], [49] and making changes to improve the stressful situation [50]. They also use escapism through substance use [48], [50], comfort eating, or trying to ignore stressful experiences [50]. There is limited research from teacher education students’ perspectives on the strategies they use to cope with stress during their educational programmes. The research available focuses predominately on coping with the practicum component. Positive strategies include seeking support [38], [51] and using leisure activities [52]. However, dysfunctional coping methods are also reported which include taking out frustrations on children [52].

The adverse impact of psychological distress upon student health, well-being and academic performance is of concern as are the maladaptive strategies used by students to cope with psychological distress. Therefore, an in-depth understanding of student psychological distress and coping is necessary to enable higher education providers to adequately support students. Consequently, the aim of this mixed method study was to determine the prevalence of psychological distress, the sources of stress and the coping processes of a sample of Irish students. Specifically, the following research questions were addressed. What is the prevalence of psychological distress among nursing/midwifery and teacher education students at one university in Ireland? What are the determinants of stress for students? What are the coping processes adopted by the students? How do students experience stress and coping? This paper adds to existing research by illuminating in depth the psychological distress experienced by undergraduate nursing/midwifery and teacher education students. Based on the results of the research, the paper makes a contribution to the field by establishing the link between student distress, ensuing maladaptive coping and lifestyle behaviours among this population. It also highlights the complex relationship between psychological distress, coping and lifestyle behaviours.

## Methods

### Study design

This mixed method research study used a Quantitative/Qualitative (Quan/Qual) explanatory two-phase design. The qualitative (interview) data were used to illuminate and build upon the initial quantitative (questionnaire) data. Combining qualitative and quantitative data helped to provide a more complete picture [53] of student stress and coping than using a single method and helped to counteract the weaknesses of each research approach [54].

### Questionnaire

A comprehensive questionnaire was created comprising two widely renowned standardized questionnaires the General Health Questionnaire (GHQ) [55] and The Ways of Coping Questionnaire (WOC) [56] as well as a questionnaire developed specifically for the study entitled the Lifestyle Behaviour Questionnaire (LBQ).

Psychological distress levels were measured through the GHQ 28 item self-report measure which is a widely used measure of psychological distress. Reliability coefficients have ranged from 0.78 to 0.95 in various studies [57]. Cronbach’s alpha coefficient in this study was 0.91. Each item has four likert response options, typically being ‘not at all’, ‘no more than usual’, ‘rather more than usual’ and ‘much more than usual’. The binary scoring method (0, 0, 1, 1) was selected, with the total score ranging from 0–28. Scores exceeding the threshold value of 5 are classed as achieving ‘psychiatric ‘caseness’ which suggests that if such respondents presented in general practice, they would be likely to receive further attention.

The WOC was selected to identify the thoughts and actions the participants used to cope with a specific stressful situation. The WOC is a likert-type self-report instrument which consists of 50 items (plus 16 fill items) within eight empirically derived scales [56]. It measures eight types of coping; confrontive coping, accepting responsibility, distancing, escape-avoidance, self- controlling, planful problem solving, seeking social support and positive reappraisal. Reliability of the instrument was evaluated by examining its internal consistency. Cronbach’s coefficient alpha ranged from 0.61 to 0.79 [56]. In this study Cronbach’s coefficient alpha ranged from 0.61 to 0.74. Participants were asked to respond to the items by indicating the frequency with which each strategy was used in the situation with 0 indicating “not used,” 1 indicating “used somewhat,” 2 indicating “used quite a bit,” and 3 indicating “used a fair bit”. Scores were calculated by summing the respondent’s responses to the items that comprise each scale. This provided a summary of the extent to which each type of coping was used. High scores indicate that the person often used the behaviours described by that scale in coping with the stressful event.

The LBQ, designed specifically for this study comprised 37 items that included a combination of likert and closed questions. The questionnaire comprised four sections: demographic and social characteristics, diet and exercise, substance use and relationships and sexuality. The questions on demographics and social characteristics were necessary as they were not catered for in the GHQ or WOC. The lifestyle data were required to cross tabulate against the data from the GHQ and WOC. The instrument was constructed following a review of literature and from an analysis of similar instruments such as the College Lifestyle and Attitudinal Study [8] (Hope et al. 2005). Experts in the field of mental health and health promotion reviewed the instrument. A pre-test (n = 178) to determine the completion time, accuracy of instructions, clarity, the best wording of the questions and appropriate distribution procedures preceded the main study. Face validity was established by conducting interviews with students and the student feedback was used to finalise the questionnaire.

### Interviews

Interviews sought to examine in more depth the experience of the higher education students as it pertained to stress, lifestyle and coping as indicated in the questionnaire. The interview schedule was informed by a critical analysis of the literature on stress, psychological distress and coping amongst higher education students, specifically teacher education and nursing/midwifery students [33]–[42]. An open and flexible interview schedule was used. The interviews were conversational in nature as is advocated by Rapley [58]. The interview questions included: a) Tell me about what it is like to be a student b) How would you characterise your experience of being a student in terms of your health and well-being? c) Is there anything stressful about being a student? d) What is that experience like? e) How do you cope? All interviews were recorded with participants’ written consent and were transcribed verbatim.

### Sample

All current teacher education students (n = 1104) and nursing/midwifery students (n = 473) in the University of Limerick in the mid-west region of Ireland were informed of the study and invited to participate by email. Direct contact with potential participants was then made during a formally timetabled period. The students were given a brief outline of the study, together with an opportunity to ask questions, after which the questionnaire was distributed. An information sheet was provided which included the nature and purpose of the study. Participation was voluntary and confidentiality of information was assured. An email was sent to each year group to ensure that those not present on the day of distribution were afforded the opportunity to complete the survey if desired. All students were also invited to take part in the interviews.

### Ethical statement

The University of Limerick Research and Ethics Committee granted approval for the study following a review of the study design, research instruments, informed consent form and participant information sheet which included an invitation to partake in the study and which emphasized the voluntary nature of participation. Study participants provided written consent.

### Data analysis

The survey data were analysed with the assistance of the Statistical Package for Social Sciences (SPSS version 18) and the Statistical Analysis Software (SAS version 9.3). Descriptive and inferential analyses were conducted. Information on demographic and social characteristics was obtained using descriptive statistics, means, medians and standard deviations for continuous variables and frequencies for categorical variables. Cross tabulations were conducted among variables. Scores were calculated for the WOC to summarise the extent to which each type of coping was used in a particular stressful episode. As the data were normally distributed parametric tests were used. Bivariate analyses (analysis of variance) were also conducted. The researchers used alpha (α) at 0.05 and confidence interval of 95%. Subsequently multivariate analyses of the variance (MANOVA) were performed for the “escape-avoidance” subscale.

The interview data were analysed thematically according to Newell and Burnard’s [59] six stage framework. Memo style notes were made after each interview. Following this the interviews transcriptions were read and reread to increase familiarity with the data and to identify general themes. Open coding was first employed with initial codes written in the transcript margin to summarise and categorise what was being said. Subsequently, where categories were overlapping similar ‘open codes’ were merged to form higher order codes. This resulted in a reduced list of codes. These codes were checked against the interview text again to ensure they accurately represented what was being said and were subsequently verified by an independent researcher. Data excerpts that embodied each theme are represented in the results section of this paper. The qualitative data were used to illuminate the quantitative data. In particular the analysis of the interview data facilitated the interpretation of the survey data related to the determinants of stress and the strategies used to cope with stress.

## Results

In total 1112 from the 1557 students completed the survey yielding a response rate of 71% (86% from nursing and 64% from education). Fifty nine students indicated their desire to participate in interview. By the fortieth interview data saturation was achieved. The researcher was sensitive to the fact that the students had volunteered to participate and that in doing so had expressed a desire to share their experiences of stress. Knowing that there can often be reluctance among students to discuss issues such as stress and psychological distress, the researcher wished to honour their desire to participate and therefore interviewed all fifty nine volunteers.

### Demographics

Table 1 presents the demographic profile of the subset of respondents included in the bivariate and multivariate analysis (n = 1030) as respondents with incomplete WOC data (n = 82) were excluded from this analysis. As evident in Table 1 the sample comprised more females (54.85%) than males (44.15% males). The majority (90.39%) of the respondents were under the age of 26 years. Most of the respondents were born in Ireland (93.11%) with the remainder born in other European counties (4.08), America (1.46%), Africa (0.49%), Asia (0.49%) and Oceania (0.19%). The students were registered on nursing/midwifery programmes (37.96%) and teacher education programmes (62.04%). During the academic year over half of the students resided in designated student accommodation both on campus (23.59%) and off campus (29.03%). Others resided in non-student rented accommodation (22.43%), the family home (17.18%) or their own homes (7.18%). The respondents indicated that their income during the academic year came from varied sources. Family, part-time work (<30 hours per week) and student grants were the most frequently reported sources of income (see Table 1).

**Table 1:** Demographic profile of sample.

Name	Categories	n	%
**Gender**	Male	465	45.15
	Female	565	54.85
**Age**	17–26	931	90.39
	27–36	76	7.38
	37–46	19	1.84
	47+	4	0.39
**Marital status**	Single	922	89.51
	Married	32	3.11
	Divorced	6	0.58
	Separated	2	0.19
	Cohabiting partner	45	4.37
	Other	23	2.23
**Country of birth**	Ireland	959	93.11
	Rest of Europe	42	4.08
	America	15	1.46
	Asia	5	0.49
	Africa	5	0.49
	Oceania	2	0.19
	Unknown	2	0.19
**Course**	Nursing/Midwifery	391	391
	Education	639	639
**Residence**	On campus studentaccommodation	243	23.59
	Off campus studentaccommodation	299	29.03
	Other rentedaccommodation	231	22.43
	Family home	177	17.18
	Own home	74	7.18
	Other	6	0.58
**Source of income**	Part time work(<30 hours per week)	428	41.55
	Full time work(>30 hours per week)	28	2.78
	Grant	283	27.48
	Family	481	46.7
	Internship (nursing students)	14	1.36
	Other	69	6.7

This table presents the demographic profile of the subset of the sample (n = 1030) included in the bivariate and multivariate analysis. It excludes respondents (n = 82) for whom incomplete data on the WOC was available.

### Determinants of stress

Analysis of the GHQ yielded that 41.9% of respondents had a score >5 which indicates significant psychological distress. The mean GHQ score was 4.89 (SD = 5.07) and the scores ranged from 0–28. The sources of this distress were varied but centred predominately on aspects of their studies and their financial, living and social pressures. Academic related stressors included exams (74.4%), assignments (71.0%), workload (67.9%), practice placements (36.7%), lectures (8.3%) and lecturers/teachers (6.6%). Other stressors included finances (51.7%), commuting to college (13.6%), being away from home (11.0%), sharing accommodation (7.2%) and making new friends (6.7%). The qualitative data reflected these determinants of stress and during the interviews the students were quite forthcoming about how they experienced these stressors. For data excerpts that illuminate these determinants see Table 2.

**Table 2:** Determinants of stress.

Finances	Workload	Exams and Assignments
*All the money worries that you have as a student… ….I* *have to pay all the fees….It very hard especially the way* *things are now. …I don’t know if I‘ll be able to go into* *third or fourth year yet (interview 22).*	*It’s difficult sometimes…last week I…I found myself being up* *until 3 or 4 o’clock in the morning trying to finish off things and* *then you go into Labs during the day and you’re just wrecked….* *Nothing is going in and then when you try to reproduce the stuff* *….you found that you haven’t really learned a huge amount. (Interview 12)*	*Presentations are very stressful…I had mine this day last week and for the week before I was waking up in a sweat over it … I get really, really bad panic attacks…. I actually had to start taking medication for it… college was a trigger I am not good with dealing with stress… I love college but it is stressful but if I didn’t love it I probably would have given it up because it made me not feel well. (Interview 56)*
*I’m working two jobs at the moment to give myself money* *because I know my parents don’t have it ….(Interview 12)*	*I’m doing a very heavy course… a lot of hours, and a lot of* *time. It’s very, very stressful at times, like really, really stressful* *at times and it’s not just me, everyone on the course trying to get* *things in, meeting deadlines, you just feel like you just don’t* *have enough hours in the day (Interview 2)*	*I find around exam times it’s stressful, the guilt of trying to manage the time you need for your exams and trying to manage home life as well…. in the couple of weeks coming up to exams I have the stress of the exams and the stress of feeling so guilty that I feel the kids are practically driving themselves around (interview 34)*
**The social life**	**Practicum placement**	**Lecturers**
*It’s a stress because it’s not like your social life at home* *where you might go out at ten or half ten and come back* *at half one or two. You enjoy actually being around your* *friends whereas I find it’s really drink as much as you can* *go to the night club, fall around the place and come home* *and drink a little bit more and maybe fall into bed at half* *five and then don’t get up all day (Interview 19)*	*Patient death is stressful …the first time it is quite shocking …. I* *wasn’t really sure what to do or how to react….we recently had* *two patients successfully commit suicide…. I went over in my head* *everything I said to him and everything he said to me just trying* *to figure out what could have been done differently or if anything* *could have been done differently…I felt a bit like I had failed the* *patient… (Interview 40).*	*My FYP my tutor was a big source of stress because I didn’t find him helpful… I did not have a notion how to analyse any of my results and he wasn’t any help for me… he just said look it up on you tube or Google to find out how to do it. There was another guy a post grad that was working with him that helped me and only for him I’d still be trying to do my FYP at this stage* (Interview 26)
*I hadn’t drunk (alcohol) beforehand and I had it in my* *mind that I wasn’t going to drink … I probably did give* *into peer pressure and did what everyone else is* *doing(Interview 57)*	*Teaching practice is definitely the most stressful part I think. I* *found it the most stressful…. Just the workload…it’s* *just constant (Interview 3)*	*I had a Lecturer there last year, he really stressed me out big time……He failed a lot of the course …I thought he was just acting up getting on a power trip…. it was stressful enough…. I didn’t know if I was going to be able to go on teacher practice (Interview 25)*
**Sharing accommodation/commuting to college**	**Making new friends**	**Being away from home**
*Sharing accommodation was stressful this year because of* *my roommate…we’re kind of not very compatible. I like to* *have my apartment really clean and tidy and he feels like* *oh I’m not living at home anymore, I can do what I want* *… I wasn’t able to confront him about it (interview 27)*	*You make the friends that you make for life in college so I was* *like on the first day oh my god if I don’t meet any friends, that* *was one of my worries…because everyone says you meet your* *partner in college as well there was a bit of stress there* *like…(interview 31)*	*The feeling that you don’t have anyone looking after you, it’s just yourself that’s looking after you are stressful at times…. I go through periods of two or three weeks and I’m very down and I don’t like talking to nobody and I stay in my room…. I’m just missing home (Interview 27)*
*If I’m having a stressful day I find the car stressful…* *(Interview 7)*	*I think it’s kind of the impersonal nature of college which makes* *you feel like you are on your own which makes you stressed more* *(Interview 45).*	*You live to go home on the Friday evening for the two days … a few days (in college) seems like ages when you are kind of struggling to get by on a daily basis. I found it pretty hard this year (Interview 19)*

This table includes excerpts from participant interviews which reflect the main stressors as experienced by the students.

### Ways of Coping

Table 3 provides a descriptive analysis (mean and standard deviation) of the results from the subscales of the WOC. High scores indicate that the person often used the behaviours described by that scale in coping with the stressful event. Table 3 also presents the key findings from the bivariate analysis which illustrates statistically significant differences in coping processes in relation to a number of demographic variables including gender, age, marital status, place of residence, programme of study, and year of study. Females used escape avoidance and seeking social support more frequently than males. Students under the age of 26 years used escape avoidance frequently in contrast to those aged 26 years and older who used positive reappraisal. Marital status was linked with the coping strategies used. Those categorised as single, divorced and separated used escape-avoidance, distancing and self- controlling frequently compared to those categorised as married, cohabiting or other. Differences in the coping processes of student nurses/midwives and teacher education students emerged. Nursing/midwifery students used escape avoidance, sought social support and positive reappraisal more frequently than teacher education students. Those residing in student accommodation used escape avoidance, distancing and self- controlling more than those living in other types of accommodation. Those who perceived that being a student was stressful used escape avoidance, accepting responsibility and confrontive coping more than those who did not.

**Table 3:** Coping strategies and factors.

			Ways of Coping Subscales [Mean (SD)]															
			ConfrontiveCoping 5.90(3.54)		AcceptingResponsability5.10 (2.90)		Distancing7.02 (3.80)		EscapeAvoidance7.92 (4.98)		Self-Controlling9.05 (4.03)		Planfulproblemsolving 7.73(3.76)		SeekingSocial Support7.09 (3.98)		PositiveReappraisal6.97 (4.28)	
Name	Categories	n	Mean	P-value	Mean	P-value	Mean	P-value	Mean	P-value	Mean	P-value	Mean	P-value	Mean	P-value	Mean	P-value
Gender	Male	465	5.88	0.8771	4.98	0.2637	6.97	0.7346	**7.47**	**0.0078**	8.79	0.0563	7.70	0.7848	**6.27**	**<0.0001**	6.99	0.9332
	Female	565	5.92		5.19		7.05		**8.30**		9.27		7.76		**7.76**		6.96	
Age	17–26	931	5.96	0.1168	5.11	0.5963	7.07	0.1925	**8.04**	**0.0169**	9.09	0.2449	7.70	0.3603	7.09	0.9187	**6.89**	**0.0442**
	>26	99	5.37		4.95		6.55		**6.79**		6.68		8.06		7.05		**7.80**	
Marital status^a^	Single	930	5.94	0.2533	5.09	0.7605	**7.10**	**0.0268**	**8.06**	**0.0075**	**9.14**	**0.0382**	7.74	0.8621	7.08	0.6219	6.97	0.8534
	Married/Other	100	5.52		5.18		**6.22**		**6.66**		**8.26**		7.67		7.15		7.05	
Programme	Nursing/Midwifery	391	6.15	0.0782	5.31	0.0581	7.08	0.6754	**8.45**	**0.0084**	9.30	0.1218	7.89	0.2836	**7.86**	**<0.0001**	**7.33**	**0.0360**
	Education	639	5.75		4.96		6.98		**7.61**		8.90		7.63		**6.62**		**6.76**	
Year of Study	Year 1	269	6.02	0.1416	**5.39**	**0.0483**	7.22	0.1621	**8.33**	**0.0061**	**9.57**	**0.0102**	7.91	0.7046	7.12	0.1842	**7.59**	**0.0051**
	Year 2	229	5.42		**4.85**		6.52		**6.91**		**8.38**		7.54		6.60		**6.22**	
	Year 3	233	6.05		**5.32**		7.09		**8.21**		**9.21**		7.64		7.31		**7.08**	
	Year 4	299	6.06		**4.85**		7.15		**8.10**		**8.97**		7.78		7.27		**6.92**	
I enjoy my course^b^	Agree	888	5.85	0.2069	5.03	0.0684	6.94	0.1197	7.81	0.0617	9.03	0.6797	7.77	0.4715	7.17	0.1082	7.03	0.2599
	Disagree/Neutral	142	6.25		5.51		7.48		8.65		9.18		7.52		6.59		6.60	
Being a student is stressful	Yes	361	**6.50**	**0.0001**	**5.58**	**<0.0001**	7.19	0.2613	**9.10**	**<0.0001**	9.38	0.0585	7.79	0.7058	7.26	0.3187	7.17	0.2718
	Sometimes + No	669	**5.59**		**4.83**		6.92		**7.29**		8.88		7.70		7.00		6.87	
Residence	Student accommodation	542	6.01	0.3369	5.23	0.1064	**7.27**	**0.0233**	**8.34**	**0.0043**	**9.34**	**0.0156**	7.74	0.9308	7.01	0.4960	6.96	0.8806
	All other	488	5.79		4.94		**6.74**		**7.46**		**8.73**		7.72		7.17		7.00	
Diet	Very healthy + Healthy	754	5.83	0.2419	**4.98**	**0.0257**	7.05	0.6348	**7.54**	**<0.0001**	8.95	0.1699	7.79	0.4402	7.16	0.3871	7.11	0.0916
	Not healthy + Unsure	273	6.12		**5.44**		6.93		**8.99**		9.34		7.59		6.92		6.60	
Eating pattern has changed^b^	Agree	694	**6.10**	**0.0107**	**5.36**	**<0.0001**	7.16	0.0737	**8.39**	**<0.0001**	**9.31**	**0.0031**	7.86	0.1038	7.15	0.5040	6.97	0.9446
	Disagree/Neutral	336	**5.50**		**4.54**		6.71		**6.96**		**8.52**		7.46		6.90		6.99	
Physical activity	Very active + Active	760	5.88	0.5820	5.07	0.5175	7.14	0.1103	**7.63**	**0.0014**	9.01	0.4399	7.84	0.1156	7.05	0.4758	**7.19**	**0.0090**
	Not active + Unsure	268	6.01		5.20		6.71		**8.76**		9.23		7.43		7.25		**6.40**	
Smoke tobacco	Yes	178	6.03	0.5940	5.35	0.1915	7.09	0.7827	**9.10**	**0.0005**	9.37	0.2566	7.65	0.7539	7.46	0.1709	**7.74**	**0.0086**
	No	852	5.88		5.04		7.00		**7.68**		8.99		7.75		7.01		**6.81**	
Drink alcohol (days/week)	≤2	815	5.80	0.0669	5.09	0.8856	6.95	0.2817	**7.73**	**0.0090**	9.11	0.5171	7.75	0.8997	7.19	0.1644	6.94	0.4941
	≥3	209	6.31		5.12		7.27		**8.74**		8.90		7.71		6.76		7.16	
Use cannabis past 6 months	Yes	104	5.89	0.9325	5.40	0.1837	**7.66**	**0.0365**	**8.94**	**0.0256**	9.15	0.7713	7.79	0.8202	6.57	0.1789	6.38	0.1513
	No	780	5.92		5.00		**6.83**		**7.77**		9.03		7.70		7.12		7.02	

This table provides a descriptive analysis (mean and standard deviation) of the results from the subscales of the WOC and key findings from the bivariate analysis which illustrates differences in coping processes in relation to demographic variables.

a Single includes divorced and separated; Married includes cohabiting.

b Agree includes both strongly agree and agree; Disagree includes both strongly disagree and disagree.

There was a statistically significant relationship between diet and escape avoidance. Those who rated their diet as “not healthy” or “unsure” and who strongly agreed or agreed that their eating pattern had changed since starting college used escape avoidance regularly. The changes involved eating more food generally and specifically eating more fried food, more carbohydrates and convenience foods and less fruit and vegetables. Those who indicated that their eating habits were influenced by mood, money, stress, worry, boredom or drinking alcohol used escape avoidance frequently. Similarly those who were current tobacco smokers, those who used cannabis in the previous six months, those who drank alcohol more than 3 days per week plus those who rated their level of physical activity as “not active” or “unsure” used escape avoidance frequently.

**Figure 1:**
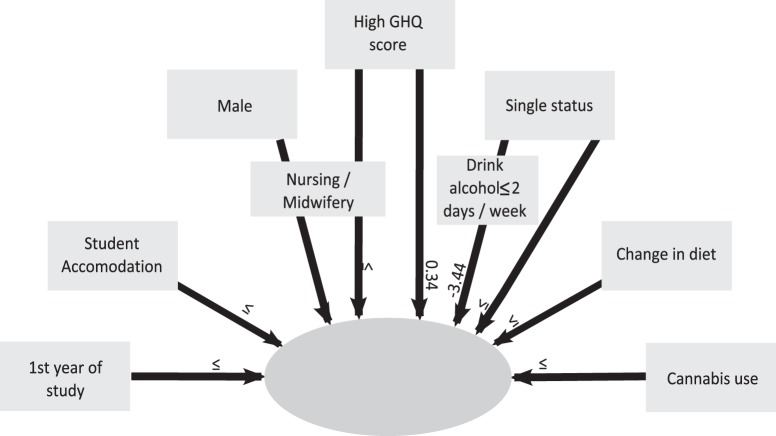
MANOVA of factors linked with escape-avoidance. This figure shows factors linked with escape- avoidance behaviours. The MANOVA coefficients are included in arrows. A positive coefficient indicates that people more frequently used escape-avoidance behaviours. A negative coefficient indicates that people less frequently used these behaviours.

A Multivariate analysis (MANOVA) of “escape-avoidance” was conducted (r2 = 0.24) (see Fig. 1). Individual factors (marital status, year of study), psychological status (GHQ score), social factors (place of residence) and lifestyle behaviours (diet, substance use) are linked to “escape avoidance”. In addition, the interaction of GHQ score and programme of study, marital status and drinking habits, gender and programme of study were linked to “escape-avoidance”.

### Strategies used to cope with stress

The interview data illuminated in more depth the coping strategies that students employed when stressed. Analysis revealed three recurrent themes, escape/avoidance, seeking support and taking control.

#### Escape/Avoidance

Students frequently attempted to escape from or avoid facing up to the stress experienced. They did this through substance use, comfort eating, isolation and denial. Student used substances such as alcohol, tobacco and cannabis to cope.

*I’d drink or I’d smoke…people smoke when they’re stressed because it is very calming…I turned to drink very, very heavily …all spirit based… I craved the kind of calmness of cannabis or something like that…. I just wanted to be that relaxed… I need something that is in some way liberating from what was going on at the time (Interview 9).*

Comfort eating was common.

*When I am stressed out I have an awful tendency to go to the chipper, my comfort food (Interview 54).*

*Whenever I get stressed I just want to eat more (interview 42).*

Isolation from others and extra time spent sleeping were means of coping for some.

*The way I like to deal with it is to be on my own (Interview 7).*

*Going to my room….not talking to anybody (Interview 27).*

*There are times when I say I just can’t cope with this today I am taking the day to myself to sleep” (Interview 54).*

Denial of stress was common with students not wanting to admit vulnerability to themselves or others.

*Stress is a kind of thing that people don’t want to admit to. You know you don’t want to seem weak….people don’t want to talk to their friends because they want to seem like they’ve everything sorted, they won’t talk to a counsellor because there’s a stigma attached to it and no one wants to talk to their parents about it, they’re not going to go to their parents and say I’m stressed out, same with their peers there’s going to be a pressure to stay on top of everything (Interview 5).*

The fear of admitting vulnerability resulted in attempts at self-protection through concealment.

*I’d hide it from everyone here (Interview 30)*

*I put a bit of a façade up in public like I’m not really that stressed (Interview 2).*

#### Seeking support

Students coped through seeking informal, formal and spiritual support. Informal support was the preferred option of most students who relied on their friends or family.

*I have some really good friends at the end of the day if I ever get too stressed or if I ever feel really overwhelmed and they just calm me down and just tell me that everything is going to be ok (Interview 10).*

*I would talk to my parents, like my mam (Interview 3).*

A minority of students disclosed the value of professional support.

*Talking over in the counselling has been great. …. just allowing stuff to come out of your head and let it walk around a bit and see how it feels I find that great (Interview 29).*

*I actually had to start taking medication ….it’s been very, very stressful but medication has changed my life (Interview 56).*

Some students sought spiritual support through prayer when faced with stressful situations.

*I pray and hope it goes well….I pray a lot (Interview 8).*

Conversely, some students actively avoided seeking support in efforts not to burden others

*I don’t like to burden other people so I just deal with it myself (Interview 2)*

#### Taking control

This theme represented efforts at confronting and dealing with the stressful situation. This included reflecting on the determinants of stress, accepting responsibility, problem solving, and putting strategies in place to prevent or manage the stress.

Students used problem solving skills including reflecting on the stressful situation and considering options to address the stressor.

*Self-reflection and looking at what’s gone wrong for you and how you can fix it and that’s my own approach to it (Interview 5).*

*I just try and figure out what the cause of it was and I try and fix it (Interview 10).*

*When they announced all the grant cuts and everything, the anxiety shot through the roof ….I needed a plan then so I found a job (Interview 4).*

Reflecting on the stressful situation often resulted in changes in behaviour which reduced stress.

*I’m a lot more organised now than I used to be…. I learned from how stressed I used to get (Interview 23).*

*My means [of coping] now is to get organised (Interview 9)*

Acknowledging that their behaviour often contributed to stress prompted a tendency for self-reliance.

*I feel like if I’m stressed it’s my doing I’m not making time to do it … it’s my own fault to be honest, my own problem, I’ll deal with it (Interview 23).*

Some students used recognised stress management techniques including relaxation, deep breathing, and thought stopping to cope with stress.

*If I’m getting very bad I do “stop” I try and take a breath and just say stop (Interview 7).*

*I do deep breathing; I try to calm myself down… I would say, there’s somebody worse out there than me and that would help (Interview 16).*

Others used distraction/diversion activities including listening to music, reading, recreational activities and sport.

*When I'm stressed or whatever I always listen to music and it does help (Interview 42).*

*I read…I find that’s very good to distract me from the stress and then it calms me down” (Interview 4).*

Physical means of releasing tension were also used.

*I’ll start cleaning….I could go cleaning for three hours non-stop (Interview 33).*

*If I’m stressed, I tend to need to do something physical to get that out, and I find that swimming clears my head (Interview 35).*

Conversely, students who found physical activity relieved stress often avoided it when most stressed due to perceived time pressures.

*When you are stressed you’d be I just don’t have the time (Interview 19).*

### Discussion

The data here indicate that the students in the study clearly contended with stress and their coping mechanisms varied significantly. It is important for higher education providers to be cognizant of stressors for students and also the potential maladaptive strategies that students employ in order to mitigate their stress. This study illuminates students’ experiences of stress and coping both quantitatively and qualitatively and provides a deeper understanding of the complex nature of these issues. An appreciation of this complexity will enable higher education providers to more effectively support their students’ mental health and well-being and consequently to enhance student success and retention [26], [60].

The findings from the GHQ identified that many of the students (41.9%) reported significant psychological distress. For the reasons indicated in the introduction to this paper it is difficult to make direct comparisons with previously published research. However, it is noteworthy that the prevalence of psychological distress reported here is greater than that reported by similar populations internationally [36], [37], [40], [61]. It is important to acknowledge that psychological distress is an indicator of mental health rather than a diagnosed mental disorder. However, the literature indicates that students with high levels of psychological distress are at increased risk for long-term mental health disorders and of particular concern is that these students remain unlikely to seek help [3]. The students in this study were also reluctant users of professional support which is clearly problematic in terms of prevention and has significant implications for mental health promotion.

The Royal College of Psychiatrists [31] recognize that universities and other higher education institutions often fail to meet the mental health needs of their students. Engaging with student stress and promoting positive mental health and well-being is essential. The international ‘Healthy Universities’ initiative, for example, advocates the university’s role not only as an academic provider but also as a resource for promoting health and well-being in students, staff and the wider community through education, research, knowledge exchange and institutional practice [62]. The creation and promotion of a culture of well-being is therefore important and engaging with students’ symptoms of stress in order to proactively address both stressors and coping mechanisms is urgent and essential.

The determinants of stress for students in this study were largely related to their course (assessments, exams, assignments, and practicum) and to social and financial concerns. These stressors are reflective of those reported in the nursing and teacher education literature. Among nursing students in particular, academic commitments and financial constraints were previously identified [63] and clinical placement related stressors were detailed [34]. Similarly for teacher education students teaching practice and workload [37] academic issues and assessment of teaching [39] have previously been reported as stressful.

The participants in this study reported many programme related stressors, particularly in relation to perceived workload. This finding is consistent with previous research in similar populations [37], [63]–[65]. The perceived workload evidenced here may be related to the extent to which the students perceived the work as meaningful [66]. Some students questioned the value of specific modules and assessments which they felt added unnecessarily to their already heavy workload. Research has found that when courses are well designed the long hours of student work are not perceived as workload [67]. Third level providers must design educational programmes of quality which meet academic standards, but that does so in a manner that is at all times cognisant of student stress. Practicum placements within professional programmes can be a core stressor for students. Educators must consider their expectations of students while on practicum placement. Excessive expectations not only created stress for the participants in this study, they also negatively impacted on learning opportunities by limiting the time available to students for reflection and self-directed learning. It is acknowledged that the relationship between stress and workload is more complex than simply “more work equals more stress” [68] (page 167) so in addition to decreasing unnecessary academic overload, actually assisting students to develop skills to better manage their time and workload is also necessary.

The interviews highlighted that the economic downturn in Ireland has compounded the financial stress experienced by students. The financial uncertainty previously highlighted by Stixrud [27] was palpable in the interviews. Students in the study relayed fears of being unable to complete their programmes and raised concerns about future job prospects. For many students financial pressures were addressed by working long hours in part-time employment. This coupled with study commitments often resulted in sleep loss, which adversely impacts the body in a similar way to stress [27]. Irish research alerts us to the strong relationship between young adults’ perceived financial stress and their mental health and well-being, therefore perceived financial stress can be considered an indicator of poor well-being by those working with young people [3]. Educators must therefore be cognisant of the multitude of life stressors experienced by students which often exacerbate academic and programme related stress.

Understanding coping behaviours is also important [69] because it facilitates deeper understanding how to better support students in difficulty. The diverse coping strategies used by students in this study were significantly related to gender, age, marital status and programme of study. Positive coping strategies included seeking social support that comprised talking to family, friends and peers. Seeking social support is an effective coping strategy which is positively correlated to psychological well-being [47] and minimising the adverse impact of encountered stressful situations on students’ wellbeing [49]. Social support is therefore important to promote among students and particularly for those with low levels of support. Students who lack social support as a protective factor require alternative supports to help build their resilience in order to enable them to more effectively deal with stress. Peer support has been found to improve stress and is advocated as a valid method of stress management for college students [70]. However, peer support strategies are just one facet of a broader solution that requires comprehensive engagement at an institutional level.

This research highlighted that the maladaptive coping strategies used by the population in this study can have potentially adverse consequences for their health and well-being. The finding that younger students are more likely to employ maladaptive responses to their stress is in keeping with the literature which indicates that younger students are more likely to ignore their emotional or mental ill health [14]. The positive relationship between GHQ score and escape avoidance behaviours including substance use (alcohol, tobacco and cannabis) and unhealthy diet is a particular concern. It is well documented that excessive stress negatively affects health behaviours [28], [71]. These include eating habits [71]; physical activity [72]; tobacco use [14], [29] and increased alcohol use [73]. It is noteworthy that behaviours that are perceived as helpful in managing stress; often conversely increase the distress. Alcohol for example is often perceived as a stress reliever but may actually make a person’s response to stress worse, conversely prolonging recovery from a stressor [74]. This finding points to the crucial nature of health education during the years spent at college, particularly as students at this stage develop habits that impact their future lifestyle choices [75]. Effective engagement with the potential adverse consequences of their maladaptive behaviours requires specific targeting from higher education providers through effective health promotion campaigns and through real engagement with the university as a setting for health promotion [76]. However, if the problem of student distress is to be comprehensively addressed, higher education providers need to critically analyse how they structure educational provision and its potential to exacerbate stress.

The population studied here are the nurses and teachers of the future. They will work in professions that are highly stressful and vulnerable to professional burnout [77], [78] which has been cited as having some genesis in the undergraduate education experience [79], [80]. Therefore, undergraduate educators in the field and higher education curricula must proactively address stress in these populations through real and meaningful engagement. As coping strategies are related to psychological well-being [41] programmes must equip students with effective coping skills which could be used in their future careers. Otherwise the cycle of distress and poor coping may follow students into their professional lives and beyond.

Recommendations include a review of current programmes to minimise the high levels of stress evidenced here particularly in relation to workload and the perceived over emphasis on assessment. The practicum is noted by students, particularly in interviews, as a significant stressor largely because of the heavy workload and also the emotional demands of working with people who are ill or whose behaviour is challenging. While it is not possible to predict the ever-changing nature of health and education environments perhaps students could be better prepared and supported. The inclusion of preparatory life skills and stress management components into programmes for students prior to practicum placements in stressful professions should be core to educational preparation rather than an optional extra. The provision of supports in ways that are both accessible and acceptable to students is necessary. Rather than a normative prescription of support by service providers, further research that examines student perspectives and needs with regard to psychological distress and lifestyle is warranted. The voices of students are essential here in order to provide effective support that students will be comfortable to access. Greater recognition of the stressful nature of being a student in higher education is needed, rather than the current view that it is a normal part of student life. Acceptance of stress as a normal part of student life denies the real and problematic situation that a significant number of students are experiencing psychological distress. Real recognition of the deleterious impact of student stress is needed in order to foster more proactive engagement with student stress by higher education providers. This engagement needs to be underpinned by cultures of prevention and health promotion.

With regard to strengths and limitations of this study, the main strengths are the large sample, the high response rate, the mixed method design and the use of previously validated instruments (GHQ and WOC). Limitations include use of a single site which results in contextual findings which may not be representative of all students. A further limitation is the use of self-report measures which are prone to socially desirable answers. As this is a cross-sectional study causalities may not be inferred and the results may not be predictive of the longitudinal relationship between self-reported psychological distress and coping. A longitudinal study to follow the students in their subsequent years to explore the pattern of changes over the duration of a programme of study would be beneficial.

## References

[1] Gore F, Bloem PJN, Patton GC, Ferguson J, Joseph V, et al (2011) Global burden of disease in young people aged 10–24 years: a systematic analysis. Lancet 377:2093–2102. 21652063 10.1016/S0140-6736(11)60512-6

[2] Blanco C, Okuda M, Wright C, Hasin DS, Grant BF, et al. (2008) Mental health of college students and their non–college-attending peers: results from the national epidemiologic study on alcohol and related conditions. Arch Gen Psychiatry 65∶1429–1437. 10.1001/archpsyc.65.12.1429 PMC2734947 19047530

[3] Dooley B, Fitzgerald A (2012) My world survey: National study of youth mental health in Ireland. Dublin: Headstrong and University College Dublin School of Psychology.

[4] Vàzquez F, Otero P, Diàz O (2012) Psychological distress and related factors in female college students. J Am Coll Heal 60:219–225. 10.1080/07448481.2011.587485 22420699

[5] Stallman HM (2010) Psychological distress in university students: a comparison with general population data. Aust Psychol 45:249–257.

[6] Verger P, Combes JB, Kovess-Masfety V, Choquet M, Guagliardo V, et al (2009) Psychological distress in first year university students: socioeconomic and academic stressors, mastery and social support in young men and women. Soc Psychiatry Psychiatr Epidemiol 44:643–650. 19096741 10.1007/s00127-008-0486-y

[7] Dyrbye LN, Thomas MR, Shanafelt TD (2006) Systematic review of depression, anxiety, and other indicators of psychological distress among U.S. and Canadian medical students. Acad Med 81:354–373. 16565188 10.1097/00001888-200604000-00009

[8] Nerdrum P, Rustøen T, Rønnestad MH (2006) Student psychological distress: a psychometric study of 1750 Norwegian 1st year undergraduate students. Scan J Educ Res 50:95–109.

[9] Humphris G, Blinkhorn A, Freeman R, Gorter R, Hoad-Reddick G, et al (2002) Psychological stress in undergraduate dental students: baseline results from seven European dental schools. Eur J Dent Educ 6∶22–29. 11872070 10.1034/j.1600-0579.2002.060105.x

[10] Bayram N, Bilgel N (2008) The prevalence and socio-demographic correlations of depression, anxiety and stress among a group of university students. Soc Psychiatry Psychiatr Epidemiol 43∶667–672. 18398558 10.1007/s00127-008-0345-x

[11] Royal College of Psychiatrists (2011) Mental health of students in higher education. (Council Report CR166). London: Royal College of Psychiatrists.

[12] Stallman M (2008) Prevalence of psychological distress in university students: implications for service delivery. Aust Fam Physician 37:673–677. 18704221

[13] Houghton F, Keane N, Murphy N, Houghton S, Dunne C (2010) Tertiary level students and the mental health index (MHI-5) in Ireland. Irish J Applied Soc Studies 10:1 Available: http://arrow.dit.ie/ijass/vol10/iss1/7 Accessed May 1^st^ 2014.

[14] Hope A, Dring C, Dring J (2005) College lifestyle and attitudinal national (CLAN) survey. Galway: National University of Ireland.

[15] Saïas T, du Roscoät E, Véron L, Guignard R, Richard JB, et al (2014) Psychological distress in French college students: demographic, economic and social stressors. Results from the 2010 National Health Barometer. BMC Public Health 4∶256. Available: http://www.biomedcentral.com/1471-2458/14/256. 10.1186/1471-2458-14-256 PMC3995499 24629002

[16] Bíró E, Ádány R, Kósa K (2011) Mental health and behaviour of students of public health and their correlation with social support: a cross sectional study. BMC Public Health 11∶871. Available: http://www.biomedcentral.com/1471-2458/11/871. 10.1186/1471-2458-11-871 PMC3271050 22087581

[17] Drapeau A, Marchand A, Beaulieu-Prévost D (2012) Epidemiology of psychological distress. In Mental Illnesses - understanding, prediction and control LAbate L, editor. INTECH, 105–134 Available: http://www.intechopen.com/books/mental-illnesses-understanding-prediction-and-control/epidemiology-of-psychological-distress.

[18] Horwitz AV (2007) Distinguishing distress from disorder as psychological outcomes of stressful social arrangments. Health 11∶273–289. 17606693 10.1177/1363459307077541

[19] Wheaton B (2007) The twain meets: distress, disorder and the continuing conundrum of categories (comment on Horwitz). Health 11∶303–319. 17606696 10.1177/1363459307077545

[20] Barlow D, Durand V (2005) Abnormal psychology: An integrative approach. Belmont, CA: Thomson Wadsworth.

[21] Adams TB, Wharton CM, Quilter L, Hirsch T (2008) The association between mental health and acute infectious illness among a national sample of 18- to 24-year-old college students. J Am Coll Health 56:657–664. 18477521 10.3200/JACH.56.6.657-664

[22] Kessler RC, Amminger GP, Aguilar-Gaxiola S, Alonso J, Lee S, et al (2007) Age of onset of mental disorders: a review of recent literature. Curr Opin Psychiatry 20:359–364. 17551351 10.1097/YCO.0b013e32816ebc8c PMC1925038

[23] Ridner SH (2004) Psychological distress: concept analysis. J Adv Nurs 45:536–545. 15009358 10.1046/j.1365-2648.2003.02938.x

[24] Wichianson JR, Bughi SA, Unger JB, Spruijt-Metz D, Nguyen-Rodriguez ST (2009) Perceived stress, coping and night-eating in college students. Stress Health 25:235–240.

[25] Lazarus RS, Folkman S (1984) Stress, appraisal and coping. New York: Springer Publishing Company.

[26] Vaez M, Laflamme L (2008) Experienced stress, psychological symptoms, self-rated health and academic achievement: a longitudinal study of Swedish university students. Soc Behav Personal 36:83–196.

[27] Stixrud WR (2012) Why stress is such a big deal. J Manage Educ 36: 135-142.

[28] Nguyen-Michel ST, Unger JB, Hamilton J, Spruijt-Metz D (2006) Associations between physical activity and perceived stress/hassles in college students. Stress Health 22:179–188.

[29] Sun J, Buys N, Stewart D Shum D (2011) Mediating effects of coping, personal belief, and social support on the relationship among stress, depression, and smoking behaviour in university students. Health Educ 111:133–146.

[30] MacGeorge E, Samter W, Gillihan S (2005) Academic stress, supportive communication and health. Commun Educ 54:365–372.

[31] Royal College of Psychiatrists (2003) The Mental Health of Students in Higher Education (Council Report CR112). London: Royal College of Psychiatrists.

[32] Morrison R, O’Connor RC (2005) Predicting psychological distress in college students: the role of rumination and stress. J Clin Psychol 61:447–460. 15468342 10.1002/jclp.20021

[33] Watson R, Gardiner E, Hogston R, Gibson H, Stimpson A, et al (2008) A longitudinal study of stress and psychological distress in nurses and nursing students. J Clin Nurs 18:270–278. 10.1111/j.1365-2702.2008.02555.x 19120753

[34] Cilingir D, Gursoy AA, Hintistan S, Ozturk H (2011) Nursing and midwifery college student’s expectations of their educators and perceived stressors during their education: a pilot study in Turkey. Int J Nurs Pract 17:486–494. 21939480 10.1111/j.1440-172X.2011.01965.x

[35] Reeve KL, Shumaker CJ, Yearwood EL, Crowell NA, Riley JB (2013) Perceived stress and social support in undergraduate nursing students’ educational experiences. Nurse Educ Today 33:419–424. 23246284 10.1016/j.nedt.2012.11.009

[36] Pryjmachuk S Richards DA (2007) Predicting stress in pre-registration nursing students. Brit J Health Psychol 12: 1∶125–44. 10.1348/135910706X98524 17288670

[37] Chaplain RP (2008) Stress and psychological distress among trainee secondary teachers in England. Educ Psychol: Int J Exp Educ Psychol 28:195–209.

[38] Gardner S (2010) Stress among prospective teachers: A review of the literature. Austral J Teacher Educ doi: 10.14221/ajte.2010v35n8.2.

[39] Montgomery L, MacFarlane D, Trumpower D (2012) Student teacher stress and physical exercise. ASBBS conference proceedings 19:974–992 Available: http://asbbs.org/files/ASBBS2012V1/PDF/M/MontgomeryC.pdf Accessed 2014 May 4.

[40] Chan DW (2002) Stress, self-efficacy, social support and psychological distress among prospective Chinese teachers in Hong Kong. Educ Psychol 22: 5∶557–60.

[41] Gustems-Carnicer J, Calderón C (2013) Coping strategies and psychological well-being among teacher education students. Eur J Psychol Educ 28:1127–1140.

[42] Jones MC, Johnston DW (2000) Reducing distress in first level student nurses: a review of the applied stress management literature. J Adv Nurs 32:66–74. 10886436 10.1046/j.1365-2648.2000.01421.x

[43] Folkman S, Lazarus RS (1980) An analysis of coping in a middle-aged community sample. J Health Soc Behav 21:219–239. 7410799

[44] Chambers GN, Roper T (2000) Why students withdraw from initial teacher training. J Educ Teaching: Int Res Pedagogy 26:25–43.

[45] Pabiton CP (2007) Problems and coping strategies of university students: Implication for counseling centres. Philippine J Couns Centres 9:78–95.

[46] Iwasaki Y (2003) Roles of leisure in coping with stress among university students: a repeated-assessment field study. Anxiety Stress Coping 16:31–57.

[47] Chao RCL (2012) Managing perceived stress among college students: The roles of social support and dysfunctional coping. J Coll Couns 15:5–21.

[48] Timmins F, Coroon AM, Byrne G, Mooney B (2011) The challenge of contemporary nurse education programmes. Perceived stressors of nursing students: mental health and related lifestyle issues. J Psychiatr Ment Health Nurs 18:758–766. 21985678 10.1111/j.1365-2850.2011.01780.x

[49] Luo Y, Wang H (2009) Correlation research on psychological health impact on nursing students against stress, coping way and social support. Nurse Educ Today 29:5–8. 18692281 10.1016/j.nedt.2008.05.019

[50] Tully A (2004) Stress, sources of stress and ways of coping among psychiatric nursing students. J Psychiatr Ment Health Nurs 11:43–47. 14723638 10.1111/j.1365-2850.2004.00682.x

[51] Murray-Harvey R (2001) How teacher education students cope with practicum concerns. Teach Educ 37:117–132.

[52] Mapfumo JS, Chitsiko N, Chireshell N (2012) Teaching practice generated stressors and coping mechanisms among student teachers in Zimbabwe. S Afr J Educ 32:155–166.

[53] Pommier J, Guével MR, Jourdan D (2010) Evaluation of health promotion in schools: a realistic evaluation approach using mixed methods. BMC Public Health 10:43 Available: http://www.ncbi.nlm.nih.gov/pmc/articles/PMC2824736/. 20109202 10.1186/1471-2458-10-43 PMC2824736

[54] Creswell JW, Plano Clark VL (2007) Designing and conducting mixed methods research. Thousand Oaks: Sage publications.

[55] Goldberg D (1981) GHQ-28. London: GL Assessment Limited.

[56] Folkman S, Lazarus RS (1988) Manual for the Ways of Coping Questionnaire. Palo Alto, CA: Consulting Psychology Press.

[57] Jackson C (2007) The General Health Questionnaire. Occup Med 57:79.

[58] Rapley TJ (2001) The art (fullness) of open-ended interviewing: some considerations on analysing interviews. Qual Res 1:303–323.

[59] Newell R, Burnard P (2006) Research for evidence-based practice. Oxford: Blackwell.

[60] El Anasari W, Stock C (2010) Is the health and wellbeing of university students associated with their academic performance? Cross sectional findings from the United Kingdom. Int J Environ Res Public Health 7: 509-527. 20616988 10.3390/ijerph7020509 PMC2872284

[61] Pryjmachuk S, Richards DA (2008) Predicting stress in pre-registration midwifery students attending a university in Northern England. Midwifery 24:108–122. 17197062 10.1016/j.midw.2006.07.006

[62] Dooris M (2001) The health promoting university: a critical exploration of theory and practice. Health Educ 101:51–60.

[63] Timmins F, Kaliszer M (2002) Aspects of nurse education programmes that frequently cause stress to nursing students – fact-finding sample survey. Nurse Educ Today 22; 203–211. 10.1054/nedt.2001.0698 12027601

[64] Harmon D, Foubert O (2011) Eurostudent survey IV report on the social and living conditions of higher education students in Ireland 2009/2010. Dublin: Health Education Authority.

[65] Pulido-Martos M, Augusto-Landa JM, Lopez-Zafra E (2012) Sources of stress in nursing students: a systematic review of quantitative studies. Int Nurs Rev 59:15–25.

[66] Kember D (2004) Interpreting student workload and the factors which shape students’ perceptions of their workload. Stud High Educ 29:165–184.

[67] Kember D, Leung DYP (2006) Characterising a teaching and learning environment conducive to making demands on students while not making their workload excessive. Stud High Educ 31:185–198.

[68] Lindsay ED, Rogers H (2010) The relationship between reported workload, stress and employment levels in first-year engineering students. Australas J Eng Educ 16:167–181.

[69] Connor-Smith JK, Flachsbart CJ (2007) Relations between personality and coping: a meta-analysis. J Pers Soc Psychol 93:1080–1107. 18072856 10.1037/0022-3514.93.6.1080

[70] Burnard P, Binti HT, Hayes D, Edwards D (2007) A descriptive study of Bruneian student nurses’ perception of stress. Nurse Educ Today 27:808–818. 17379361 10.1016/j.nedt.2006.11.002

[71] Tavolacci MP, Ladner J, Grigioni S, Richard L, Villet H, et al (2013) Prevalence and association of perceived stress, substance use and behavioral addictions: a cross-sectional study among university students in France, 2009–2011. BMC Public Health 13∶724. Available: http://www.biomedcentral.com/1471-2458/13/724. 10.1186/1471-2458-13-724 PMC3750571 23919651

[72] Hudd S, Dumlao J, Erdmann-Sager D, Murray D, Phan E, et al (2000) Stress at college: effects on health habits, health status and self-esteem. Coll Stud J 34:217–227.

[73] Butler AB, Dodge KD, Faurote EJ (2010) College student employment and drinking: a daily study of work stressors, alcohol expectancies, and alcohol consumption. J Occup Health Psychol 15:291–303. 20604635 10.1037/a0019822 PMC2914610

[74] Childs E, O’Connor S, de Wit H (2011) Bidirectional interactions between acute psychosocial stress and acute intravenous alcohol in healthy men. Alcohol Clin Exper Res 35:1794–1803. 21762177 10.1111/j.1530-0277.2011.01522.x PMC3183385

[75] Robotham D, Julian C (2006) Stress and the higher education student: a critical review of the literature. J Further High Educ 30:107–117.

[76] Dooris M, Doherty S (2010) Healthy universities–time for action: a qualitative research study exploring the potential for a national programme. Health Promot Int 25:94–106. 20167825 10.1093/heapro/daq015

[77] Mearns J, Cain JE (2003) Relationships between teachers’ occupational stress and their burnout and distress: roles of coping and negative mood regulation expectancies. Anxiety Stress Coping 16:71–82.

[78] Ilhan MN, Durukan E, Taner E, Marval I, Bumin MA (2008) Burnout and its correlates among nursing staff: questionnaire survey. J Adv Nurs 61:100–106. 18034813 10.1111/j.1365-2648.2007.04476.x

[79] Gold Y (1985) Does teacher burnout begin with student teaching? Educ 105:254–257.

[80] Rella S, Winwood PC, Lushington K (2009) When does nursing burnout begin? An investigation of the fatigue experience of Australian nursing students. J Nurs Manage 17:886–897. 10.1111/j.1365-2834.2008.00883.x 19793246

